# Supplementary material for: Oral Corticosteroid Abuse and Self-Prescription in Italy: A Perspective from Community Pharmacists and Sales Reports before and during the COVID-19 Era
Source: J Pers Med. 2023 May 15;13(5):833. doi: 10.3390/jpm13050833 (PMC10220562; doi:10.3390/jpm13050833)
Supplement: Supplementary file 1 [file jpm-13-00833-s001.zip › jpm-2373645-supplementary.pdf]

SUPPLEMENTARY MATERIAL

Note each of the following questions was repeated twice; once as regards the pre-pandemic period and once as regards the post-pandemic period.

Question 1

“From your perception, how would you quantify patient's corticosteroid abuse? In particular, out of 100 subjects who requires systemic cortisone, how many do it without a prescription or with an expired prescription?”. The answers are reported in Table s1.

Italian version: “*Dalla sua percezione come quantificherebbe l'abuso di corticosteroidi da parte del paziente. In particolare su 100 soggetti che richiedono cortisonici sistemici quanti lo fanno senza ricetta o con ricetta scaduta?*"

Perception of OCS abuse (fraction of clients out of 100 who demand for systemic corticosteroids without a prescription or with an expired one),

Answer (fraction of clients/100)	Before Pandemic N (%)	Post Pandemic N (%)	p-value
0-10	90 (23.9%)	58 (15.4%)	**0.003**
11-20	72 (19.1%)	47 (12.5%)	**0.013**
21-30	65 (17.3%)	71 (18.9%)	0.568
31-40	36 (9.6%)	42 (11.2%)	0.473
41-50	35 (9.3%)	27 (7.2%)	0.289
51-60	29 (7.7%)	44 (11.7%)	0.065
61-70	23 (6.1%)	19 (5.1%)	0.525

71-80	12 (3.2%)	33 (8.8%)	**0.001**
81-90	12 (3.2%)	29 (7.7%)	**0.006**
91-100	2 (0.5%)	6 (1.6%)	0.155

Table s1. This table shows parmacists' answers to question 1. Differences between the pre- and post-pandemic era were assessed with a Student t-test.

Question 2

"Specify the frequency of systemic corticosteroid request without a prescription or an expired prescription in different age groups". The answers are reported in Table s2.

Italian version: "*Tra gli assistiti che richiedono cortisonici sistemici senza ricetta o ricetta scaduta le fascia di età più frequenti sono*".

Age distrubution of clients that request systemic corticosterodis without a valid prescription

Variable	Secondary Variable	Before Pandemic N (%)	Post Pandemic N (%)	p-value
0-17	Never	208 (55.3%)	173 (46%)	**0.011**
	Sometimes	118 (31.4%)	135 (35.9%)	0.190
	Often	35 (9.3%)	44 (11.7%)	0.285
	Very Often	13 (3.5%)	22 (5.9%)	0.119
	Always	2 (0.5%)	2 (0.5%)	1
18-35	Never	41 (10.9%)	36 (9.6%)	0.548
	Sometimes	212 (56.4%)	158 (42%)	**<0.001**

	Often	102 (27.1%)	126 (33.5%)	0.057
	Very Often	19 (5.1%)	49 (13%)	**<0.001**
	Always	2 (0.5%)	7 (1.9%)	0.094
36-60	Never	6 (1.6%)	8 (2.1%)	0.589
	Sometimes	118 (31.4%)	107 (28.5%)	0.381
	Often	174 (46.3%)	167 (44.4%)	0.608
	Very Often	69 (18.4%)	82 (21.8%)	0.237
	Always	9 (2.4%)	12 (3.2%)	0.420
61-74	Never	36 (9.6%)	34 (9%)	0.802
	Sometimes	192 (51.1%)	163 (43.4%)	**0.034**
	Often	104 (27.7%)	128 (34%)	0.058
	Very Often	39 (10.4%)	46 (12.2%)	0.420
	Always	5 (1.3%)	5 (1.3%)	1
75-84	Never	108 (28.7%)	96 (25.5%)	0.325
	Sometimes	193 (51.3%)	185 (49.2%)	0.560
	Often	49 (13%)	68 (18.1%)	0.060
	Very Often	21 (5.6%)	24 (6.4%)	0.644
	Always	5 (1.3%)	3 (0.8%)	0.477
85 over	Never	192 (51.1%)	172 (45.7%)	0.145
	Sometimes	146 (38.8%)	154 (41%)	0.551
	Often	20 (5.3%)	33 (8.8%)	0.064
	Very Often	13 (3.5%)	12 (3.2%)	0.839

	Always	5 (1.3%)	5 (1.3%)	1

Table s2. This table shows parmacists' answers to question 2. Differences between the pre- and post-pandemic era were assessed with a Student t-test.

Question 3

Which was the distribution in percentage among patients who required systemic corticosteroids without prescription or with an expired prescription for which the drug was already dispensed multiple times in the past (chronic users) and for who were asking for the drug for the first time (first-time users). The answers are reported in Table s3.

Italian version: "*Qual è stata la distribuzione percentuale tra pazienti che richiedono i corticosteroidi sistemici senza ricetta o con ricetta scaduta tra pazienti per i quali il farmaco era stato già più volte dispensato in passato, per la medesima motivazione clinica e tra pazienti che lo richiedono per la prima volta?*".

Subdivision of clients requiring systemic corticosterodis without a valid prescription in to chronic users and first-time users.

Variable	Secondary Variable (fraction of clients/100)	Before Pandemic	Post Pandemic	p-value
Chronic users	0-10	3 (0.8%)	8 (2.1%)	0.129
	11-20	7 (1.9%)	8 (2.1%)	0.794
	21-30	14 (3.7%)	16 (4.3%)	0.710

	31-40	36 (9.6%)	36 (9.6%)	1
	41-50	56 (14.9%)	70 (18.6%)	0.172
	51-60	74 (19.7%)	72 (19.1%)	0.854
	61-70	46 (12.2%)	41 (10.9%)	0.569
	71-80	55 (14.6%)	61 (16.2%)	0.545
	81-90	56 (14.9%)	39 (10.4%)	0.062
	91-100	29 (7.7%)	25 (6.6%)	0.572
First use	0-10	80 (21.3%)	59 (15.7%)	**0.049**
	11-20	54 (14.4%)	62(16.5%)	0.419
	21-30	48 (12.8%)	42 (11.2%)	0.500
	31-40	70 (18.6%)	67 (17.8%)	0.777
	41-50	62 (16.5%)	72 (19.1%)	0.341
	51-60	35 (9.3%)	40 (10.6%)	0.543
	61-70	16 (4.3%)	16 (4.3%)	1
	71-80	6 (1.6%)	9 (2.4%)	0.434
	81-90	3 (0.8%)	5 (1.3%)	0.477
	91-100	2 (0.5%)	4 (1.1%)	0.412

Table s3. This table shows parmacists’ answers to question 2. Differences between the pre- and post-pandemic era were assessed with a Student t-test.

Question 4

Which was the distribution in percentage of the following justifications for which clients asked for systemic corticosteroids without prescription or with an expired one? The answers are reported in Table s4.

Italian version: "*In che percentuale si distribuivano le seguenti giustificazioni date dai pazienti per assenza di ricetta o ricetta scaduta inerente corticosteroidi orali?*"

Frequency of corticosteroid request without a valid prescription in depending on the justification.

Variable	Secondary Variable	Before Pandemic N (%)	Post Pandemic N (%)	p-value
Emergency use	0-10	53 (14.1%)	39 (10.4%)	0.119
	11-20	56 (14.9%)	64 (17%)	0.426
	21-30	51 (13.6%)	55 (14.6%)	0.675
	31-40	96 (25.5%)	96 (25.5%)	1
	41-50	42 (11.2%)	52 (13.8%)	0.270
	51-60	23 (6.1%)	21 (5.6%)	0.756
	61-70	20 (5.3%)	29 (7.7%)	0.184
	71-80	25 (6.6%)	10 (2.7%)	**0.009**
	81-90	5 (1.3%)	9 (2.4%)	0.281
	91-100	5 (1.3%)	1 (0.3%)	0.101
Hardship in getting the prescription	0-10	45 (12%)	24 (6.4%)	**0.008**
	11-20	43 (11.4%)	38 (10.1%)	0.556
	21-30	74 (19.7%)	53 (14.1%)	**0.041**

	31-40	98 (26.1%)	85 (22.6%)	0.269
	41-50	51 (13.6%)	72 (19.1%)	**0.038**
	51-60	29 (7.7%)	38 (10.1%)	0.249
	61-70	17 (4.5%)	26 (6.9%)	0.158
	71-80	11 (2.9%)	24 (6.4%)	**0.024**
	81-90	5 (1.3%)	5 (1.3%)	1
	91-100	3 (0.8%)	11 (2.9%)	**0.031**
Forgotten prescription	0-10	112 (29.8%)	170 (45.2%)	**<0.001**
	11-20	61 (16.2%)	57 (15.2%)	0.688
	21-30	55 (14.6%)	40 (10.6%)	0.100
	31-40	79 (21%)	69 (18.4%)	0.359
	41-50	40 (10.6%)	27 (7.2%)	0.096
	51-60	16 (4.3%)	6 (1.6%)	**0.031**
	61-70	7 (1.9%)	5 (1.3%)	0.561
	71-80	2 (0.5%)	0 (0%)	Not applicable
	81-90	2 (0.5%)	2 (0.5%)	1
	91-100	2 (0.5%)	0 (0%)	Not applicable

Table s4. This table shows parmacists' answers to question 4. Differences between these two periods were assessed with a Student t-test.

Question 5

Which were the most frequent medical conditions affecting clients who asked for systemic corticosteroids without prescription or with an expired one? The medical conditions for which pharmacists were asked to specify the frequency of systemic corticosteroids request without a valid prescription were: rheumatological disorders, upper airway diseases, obstructive airway diseases, cutaneous diseases, sore throat, allergies. The answers are reported in Table s5.

Italian version: "*Tra gli assistiti che richiedono il farmaco senza ricetta o ricetta scaduta le patologie più frequenti sono?*"

Frequency of corticosteroid request without a valid prescription in different medical conditions.

Variable	Secondary Variable	Before Pandemic	Post Pandemic	p-value
Rheumatological diseases	Never	66 (17.6%)	77 (20.5%)	0.307
	Sometimes	223 (59.3%)	198 (52.7%)	0.066
	Often	70 (18.6%)	72 (19.1%)	0.852
	Very Often	15 (4%)	25 (6.6%)	0.104
	Always	2 (0.5%)	4 (1.1%)	0.412
Upper airways diseases	Never	15 (4%)	10 (2.7%)	0.309
	Sometimes	78 (20.7%)	70 (18.6%)	0.463
	Often	157 (41.8%)	149 (39.6%)	0.553
	Very Often	112 (29.8%)	125 (33.2%)	0.308
	Always	14 (3.7%)	22 (5.9%)	0.172
	Never	10 (2.7%)	10 (2.7%)	1

Obstructive airway diseases	Sometimes	110 (29.3%)	92 (24.5%)	0.139
	Often	156 (41.5%)	141 (37.5%)	0.263
	Very Often	90 (23.9%)	114 (30.3%)	**0.049**
	Always	10 (2.7%)	19 (5.1%)	0.088
Pulmonary diseases	Never	66 (17.6%)	42 (11.2%)	**0.013**
	Sometimes	151 (40.2%)	102 (27.1%)	**<0.001**
	Often	109 (29%)	118 (31.4%)	0.474657
	Very Often	43 (11.4%)	95 (25.3%)	**< 0.00001**
	Always	7 (1.9%)	19 (5.1%)	**0.017**
Cutaneous diseases	Never	51 (13.6%)	38 (10.1%)	0.142
	Sometimes	150 (39.9%)	136 (36.2%)	0.293
	Often	104 (27.7%)	119 (31.6%)	0.231
	Very Often	63 (16.8%)	70 (18.6%)	0.504
	Always	8 (2.1%)	13 (3.5%)	0.269
Sore throat	Never	73 (19.4%)	56 (14.9%)	0.100
	Sometimes	150 (39.9%)	127 (33-8%)	0.082
	Often	99 (26.3%)	97 (25.8%)	0.868
	Very Often	42 (11.2%)	68 (18.1%)	**0.007**
	Always	12 (3.2%)	28 (7.4%)	**0.009**
Allergies	Never	62 (16.5%)	60 (16%)	0.843
	Sometimes	153 (40.7%)	146 (38.8%)	0.602
	Often	93 (24.7%)	92 (24.5%)	0.933

	Very Often	53 (14.1%)	65 (17.3%)	0.229
	Always	15 (4%)	13 (3.5%)	0.700

Table s5. This table shows parmacists’ answers to question 5. Differences between the pre- and post-pandemic era were assessed with a Student t-test.
